# Charge Carrier Mobility in Poly(N,N′-bis-4-butylphenyl-N,N′-bisphenyl)benzidine Composites with Electron Acceptor Molecules

**DOI:** 10.3390/polym16050570

**Published:** 2024-02-20

**Authors:** Alexey R. Tameev, Alexey E. Aleksandrov, Ildar R. Sayarov, Sergey I. Pozin, Dmitry A. Lypenko, Artem V. Dmitriev, Natalia V. Nekrasova, Andrey Yu. Chernyadyev, Aslan Yu. Tsivadze

**Affiliations:** Frumkin Institute of Physical Chemistry and Electrochemistry of the Russian Academy of Sciences, Leninsky Prosp., 31, Bld. 4, 119071 Moscow, Russia

**Keywords:** poly-TPD, copper (II) pyropheophorbide, polymer composite, charge carrier mobility, CELIV, SCLC, photoconductivity

## Abstract

Polymer composites based on poly(N,N′-bis-4-butylphenyl-N,N′-bisphenyl)benzidine (poly-TPD) with PCBM and copper(II) pyropheophorbide derivative (Cu-PP) were developed. In thin films of the poly-TPD and Cu-PP composites, the charge carrier mobility was investigated for the first time. In the ternary poly-TPD:PCBM:Cu-PP composite, the electron and hole mobilities are the most balanced compared to binary composites and the photoconductivity is enhanced due to the sensitization by Cu-PP in blue and red spectral ranges. The new composites are promising for use in the development of photodetectors.

## 1. Introduction

**Figure 1:**
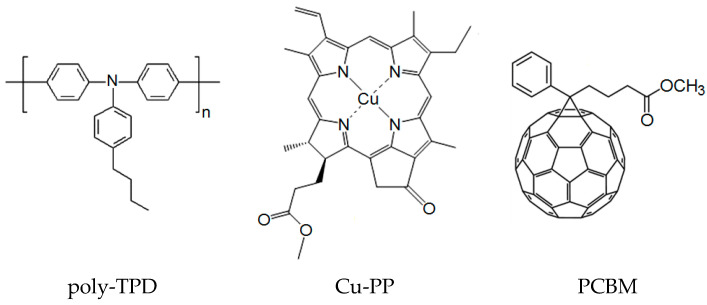
Chemical structures of materials used.

In materials design, polymers are important because their electronic properties can be controlled during both the synthesis and the creation of solid-state materials for various applications, particularly those related to organic electronics [1]. Charge carrier mobility is a key parameter characterizing the electrical properties of polymer and organic semiconductors being developed for use in electronic devices. [2]. Interest in poly(N,N′-bis-4-butylphenyl-N,N′-bisphenyl)benzidine, also known as poly-TPD (Figure 1), is caused by its ability to form thin films of high quality with excellent hole transport, i.e., monopolar electrical conductivity. The hole mobility in thin polymer layers was reported in the order of (1 ÷ 2) × 10^−3^ cm^2^V^−1^s^−1^ [3,4], 1.7 × 10^−4^ cm^2^V^−1^s^−1^ [5] and 4 × 10^−7^ cm^2^V^−1^s^−1^ [6] as extracted from the space charge limited current (SCLC) mode. In the organic field-effect transistor architecture, the hole mobility of poly-TPD was found to be 1 × 10^−4^ cm^2^V^−1^s^−1^ [7]. Possessing reasonably extended hole mobility, poly-TPD is applicable as a hole transport layer (HTL) in thin film devices due to its highest occupied molecular orbital (HOMO) energy level providing the hole transport matches well with HOMO levels of hole transport polymers and organic photoconductors. In particular, the polymer was used as an HTL in the development of organic photodiodes [8], organic light-emitting diodes (OLEDs) [4,7], quantum-dot-based light-emitting diodes (QLEDs) [5,9,10], and organic phototransistors [8]. In addition, due to the HOMO level of poly-TPD matching well with the work function of ITO and valence band of perovskite derivatives, the polymer successfully served as an HTL in perovskite solar cells [6,11,12]. Moreover, poly-TPD can exhibit photoconductivity. A blend of an electron donor (D) derivative of poly-TPD and an electron acceptor (A) PCBM (phenyl-C_61_-butyric acid methyl ester) was shown to enhance light absorption and promote the separation of electron–hole pairs in the photoactive film of an inorganic/organic heterojunction-based photodetector [13] and organic photovoltaic cell [14]. The photoconductivity spectrum of the poly-TPD:PCBM blend was additively composed of the absorption spectra of the polymer and PCBM, and to expand the photosensitive spectrum, sensitizing additives were introduced into the D-A composite. Nevertheless, the mobility of charge carriers in D-A composites based on poly-TPD remains poorly studied.

Semisynthetic macrocyclic compounds (such as porphyrins, pheophorbide-a, pheophorbide-b, chlorins, and bacteriochlorins) extracted from animal blood, plant chlorophyll or from bacterial cultures and followed by their chemical modification [15] are of great interest as active components of composite materials for research in photodynamic therapy of cancer diseases [16], water photolysis for hydrogen generation [17], converting solar energy into chemical energy [18], and electroluminescent devices [19,20]. Unlike semisynthetic coproporphyrins, mesoporphyrins, and etioporphyrins [20], pheophorbides and bacteriochlorins contain stereocenter carbon atoms associated with the aromatic cycle. Due to stereocenters, the molecules can absorb photon incidents not only at right angles to the planes of aromatic rings [21,22], but also with a significant deviation from orthogonality increasing the harvest of incident light. In addition, stereocenters in the molecules can exhibit the effect of circularly polarized luminescence, for example, similar to polymers containing chiral fragments of cholesterol [23].

In this work, the copper (II) complex with pyropheophorbide-a (Cu-PP) is used as a component of composites. The copper (II) complex with pyropheophorbide-a exhibits no luminescence in the visible range while the free base of pyropheophorbide-a demonstrates intense fluorescence in the red band of the spectrum. Therefore, we can expect that in a composite containing Cu-PP, the possible luminescent loss of absorbed light energy is not caused by the Cu-PP component. We have experience in forming a polymer composite with chlorin. Chlorin Cu-C-e6, an analog of Cu-PP, incorporated into the polymer composite exhibits electroluminescence and is applicable in light-emitting diodes [20]. In the present work, we develop composite materials based on poly-TPD with the addition of Cu-PP, which is able to enhance absorption in the blue and red ranges of the spectrum. For the first time, we study the mobility of charge carriers in a poly-TPD composite with Cu-PP and in a ternary composite based on poly-TPD, Cu-PP, and PCBM (Figure 1) using the linear increasing voltage (CELIV) technique and space charge limited current (SCLC) mode.

## 2. Materials and Methods

### 2.1. Materials and Synthesis

Poly[N,N′-bis(4-butylphenyl)-N,N′-bis(phenyl)-benzidine] (poly-TPD) from American Dye Source, Inc. (ADS254BE) (Baie-D’Urfe, QC, Canada), [6,6]-Phenyl-C61-butyric acid methyl ester (PCBM) from SES Research, C_60_ fullerene from MST (“Modern Synthesis Technology”, St. Petersburg, Russia) and bathocuproine (BCP) from Kintec (Hong Kong, China) were used as received. PEDOT:PSS (poly(3,4-ethylene-dioxythiophene):polystyrenesulfonate) from Heraeus (Clevios P VP AI 4083) (Hanau, Germany) was filtered prior to use, The ITO-coated glass (7 Ohm/□) was purchased from Kaivo (Zhuhai, Guangdong, China).

**Figure A1:**
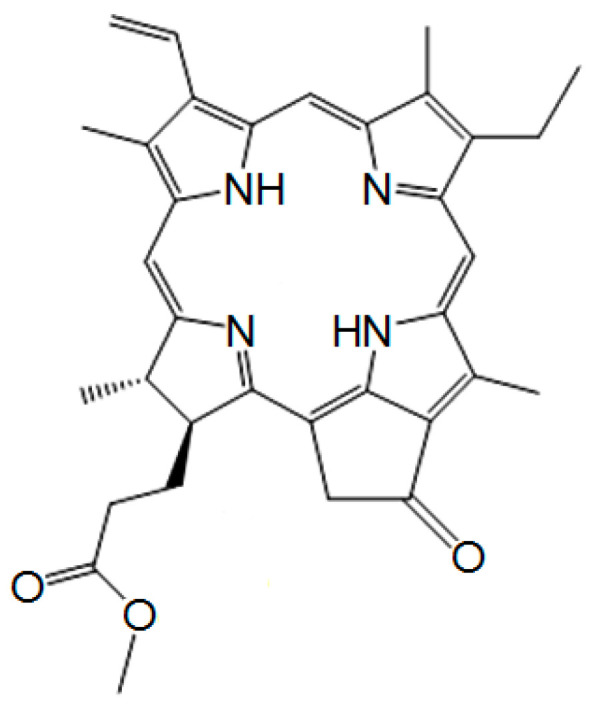
Chemical structure of free base of methyl ester of pyropheophorbide-a.

The free base of methyl ester of pyropheophorbide-a (Figure A1) was obtained according to procedure [24]. The structure and purity of the compound were confirmed by NMR, UV–Vis, and luminescent spectroscopy. For the synthesis of Cu(II) methyl ester of pyropheophorbide-a (Cu-PP) shown in Figure 1, 10 mg of the free base of methyl ester of pyropheophorbide-a was dissolved in 25 mL of methylene chloride and a solution of 7 mg of Cu(II) acetate in 15 mL of ethanol was added to the resulting solution. The solutions were mixed, and the resulting solution was stirred at 35 °C for 40 min. The solvents were removed in a vacuum. The product was dissolved in methylene chloride and purified by column chromatography from traces of the original methyl ester of pyropheophorbide-a, and the solvent was removed in vacuum. The yield of Cu-PP was 10.5 mg (95%). The structure of the resulting Cu-PP compound was confirmed by MALDI TOF mass spectrometry and UV–Vis spectroscopy in a chlorobenzene solution.

### 2.2. Cyclic Voltammetry and Energy Levels

**Figure 2:**
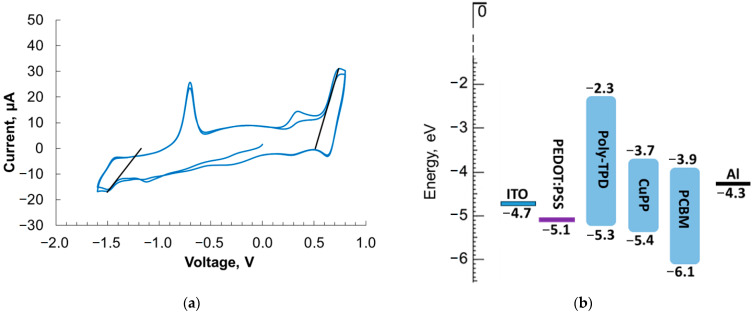
(**a**) Cyclic voltammograms of Cu-PP, the black lines show the tangents to the curves. The accuracy of the CV experiments is ±0.02 V. (**b**) Energy levels of the used materials.

Cyclic voltammetry (CV) measurements were performed for calculation of the lowest unoccupied molecular orbital (LUMO) and HOMO energy levels. The CV experiment was carried out at the scan rate of 20 mV/s in a three-electrode, three-compartment electrochemical cell in the glove box with dry argon atmosphere. Platinum sheets served as working and counter electrodes. A 0.2 M solution of tetrabutylammonium hexafluorophosphate (NBu_4_PF_6_, Fluka) (Pittsburgh, PA, USA) in acetonitrile (ACN) was used as an electrolyte. An Ag wire immersed into the electrolyte solution with the addition of 0.1 M AgNO_3_ was used as a pseudo reference electrode (Ag/Ag^+^). It was calibrated against ferrocene/ferricenium couple (−0.039 V vs. Ag/Ag^+^) and its potential was recalculated to the energy scale using −4.988 eV value for Fc/Fc^+^ in ACN. Thus, the energy level of Ag/Ag^+^ is as *E*_ref_ = −5.03 eV. The values of potentials corresponding to the HOMO and LUMO levels were determined by applying a tangent to the onset of anodic and cathodic currents (Figure 2a), with *E*_HOMO_ = *E*_ref_ − *E*_a_ (typically, *E*_a_ > 0), *E*_LUMO_ = *E*_ref_ − *E*_c_ (typically, *E*_c_ < 0).

Other details of the CV were the same as described in our previous article [19]. The obtained values of the HOMO and LUMO energy levels for Cu-PP as well as for poly-TPD and PCBM [14] are shown in Figure 2b.

### 2.3. Thin Films and Device Preparation

The mobility of charge carriers was studied in films of poly-TPD:PCBM, poly-TPD:Cu-PP, and poly-TPD:PCBM:Cu-PP composites in molar ratios of 6.33:1, 6.33:1, and 6.33:1:1, respectively.

To measure the mobility of charge carriers in the transient current mode of the CELIV technique, samples of the ITO/SiO_2_/composite/Al structure were prepared as follows. A 70 nm thick SiO_2_ layer was deposited onto a glass substrate with an electrically conductive ITO layer using magnetron sputtering. A solution of the polymer composite in chlorobenzene was deposited on top of the dielectric layer by spin-coating method at a speed of 1500 rpm, then the sample was dried in an argon atmosphere at 80 °C for 2 h. The 80 nm thick Al counter electrode was deposited by the resistive thermal evaporation (RTE) technique using an MB Evap vacuum thermal evaporator (CreaPhys, Dresden, Germany) at a vacuum of 6 × 10^−6^ mbar as described earlier [19].

To measure electron mobility in the SCLC mode, electron-only and hole-only devices were prepared with a structure of Al/composite/Al and ITO/PEDOT:PSS/composite/MoO_3_/Al, respectively. A 20 nm thick MoO_3_ layer was deposited by the RTE technique and 30 nm thick PEDOT:PSS layer was deposited by spin-coating to block electrons. Thicknesses of the poly-TPD composite layers and both Al electrodes were 75 ÷ 80 nm and 80 nm, respectively.

**Figure 3:**
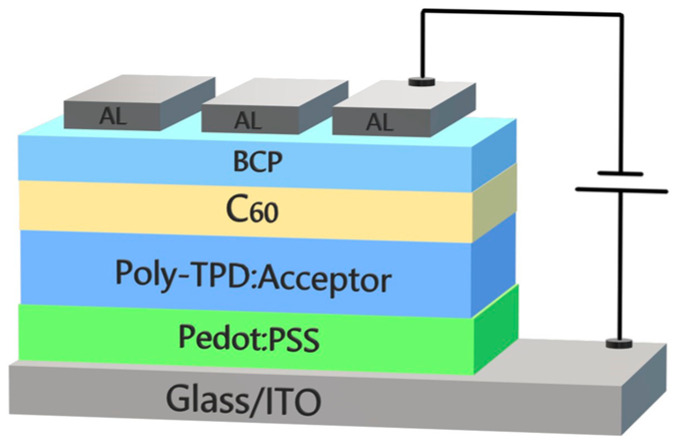
A sketch of device structure with functional layers.

The photoconductivity of the composites was investigated using ITO/PEDOT:PSS/composite/C_60_/BCP/Al devices (Figure 3), with the 30 nm thick PEDOT:PSS layer serves as a hole transport (electron blocking) layer, and the 10 nm/7.5 nm thick C_60_/BCP layers provide electron transport (hole blocking). The C_60_ and BCP layers were deposited by the RTE technique. The thickness of the composite layers ranged from 75 to 80 nm.

### 2.4. Electrical Characterization

For mobility measurements, the MIS-CELIV mode was used, which allows recording of the current of monopolar charge carriers. The experimental setup included a USB oscilloscope (DL-Analog Discovery, Digilent Co., Pullman, WA, USA), which generated master pulse and recorded transient current, as described in our works [19,20,25]. The mobility *μ* was calculated according to the expression:

$$\mu =\frac{2d^{2}}{3At_{max}^{2}\left(1+0.36\frac{∆J}{J\left(0\right)}\right)}\;,$$

here *A* is the voltage ramp, *d* is the composite film thickness, *J*(0) is the capacitance current, and Δ*J* is the conduction current at the time *t_max_*.

In the SCLC mode, the charge carrier transport occurs in the nontrapping regime and the current *J_SCLC_* obeys the Mott–Gurney equation [26]

$$J_{SCLC}=\frac{9\mu_{SCLC}\varepsilon \varepsilon_{0}V^{2}}{8d^{3}}$$

where *µ_SCLC_* is the charge carrier mobility, *ε* is the dielectric constant of the composite, *ε*_0_ is the vacuum permittivity, *V* is the applied voltage. We used *ε* = 3.5 for the composites studied.

*J*–*V* characteristics of the devices were recorded by an SMU Keithley 2400 (Solon, OH, USA, photocurrent was measured under illumination provided by a solar simulator with a 150 W Xe lamp (Newport 67005) (Newport Corp., Irvine, CA, USA). The relative error of *J*–*V* measurements was 5%. Processing of *J*–*V* curves in SCLC mode for calculation of mobility was shown earlier [19,27].

All the measurements were carried out at room temperature in an inert atmosphere of Ar with an oxygen and water content of <10 ppm.

## 3. Results and Discussion

### 3.1. UV–Vis Spectroscopy

**Figure 4:**
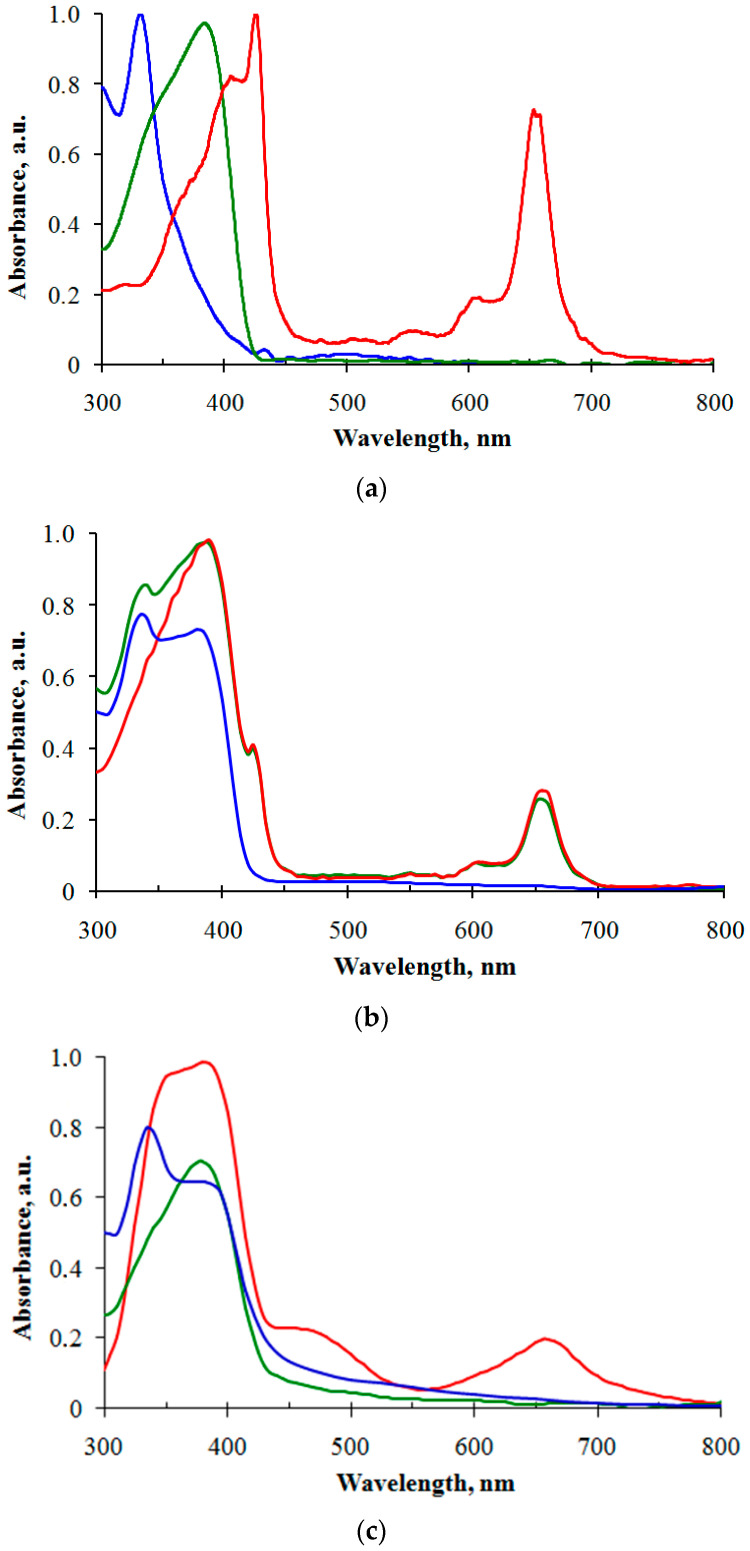
(**a**) Absorption spectra of CuPP (red), PCBM (blue), and poly-TPD (green) in chlorobenzene (10^−5^ M, optical pass length is 1 cm). (**b**) Absorption spectra of poly-TPD:CuPP (red), poly-TPD:PCBM (blue), and poly-TPD:PCBM:Cu-PP (green) in chlorobenzene solution. (**c**) Absorption spectra of poly-TPD:CuPP (red), poly-TPD:PCBM (blue) composite films, and single poly-TPD film (green).

UV–Vis absorption spectra were recorded with a Shimadzu UV-3101PC spectrophotometer (Shimadzu Corp., Kyoto, Japan). Absorption spectra of the individual components are shown in Figure 4a. PCBM exhibits typical absorption in the UV range. The UV–visible spectrum of Cu-PP exhibits a Soret band between 340 and 435 nm and a Q band between 590 and 690 nm (Figure 4a), which are characteristic of compounds such as chlorophyll A. In the absorption spectra of the composites in chlorobenzene solution presented in Figure 4b, the absorption bands of the individual components are clearly visible. The absorption of the poly-TPD (Figure 4c) is slightly different from the absorption for TPD small molecules (Figure 4a). TPD molecules are characterized by two absorption bands in the ranges of 300–330 nm and 350–390 nm, corresponding to π-π* transitions for peripheral rings and the biphenyl fragment, respectively [28]. The dominance of the latter is more pronounced for the poly-TPD than for the small molecules. This is typical for polymers with a fairly high degree of polymerization and a small number of terminal groups.

The absorption spectra of the composite films are shown in Figure 4c. The absorption spectra of the poly-TPD:Cu-PP and poly-TPD:PCBM:Cu-PP composite films are identical in the visible range. The absorption spectrum of the poly-TPD:PCBM composite film is simply the addition of the absorption bands of PCBM and poly-TPD, peaking at wavelengths around 340 and 390 nm, respectively. In contrast to the poly-TPD:PCBM composite, in the spectrum of the poly-TPD:CuPP composite film, in addition to the intrinsic absorption bands of poly-TPD and CuPP, a new absorption band appears in the range of 450–500 nm. This band reflects the donor–acceptor interaction between TPD donor and Cu-PP acceptor forming the charge transfer complex. In addition, TPD cation radicals are known to absorb in this range [28]. Thus, Cu-PP can both sensitize poly-TPD in the red spectral range and enhance the absorption of the composite in the blue–cyan range.

### 3.2. AFM of Solid Layers

**Figure 5:**
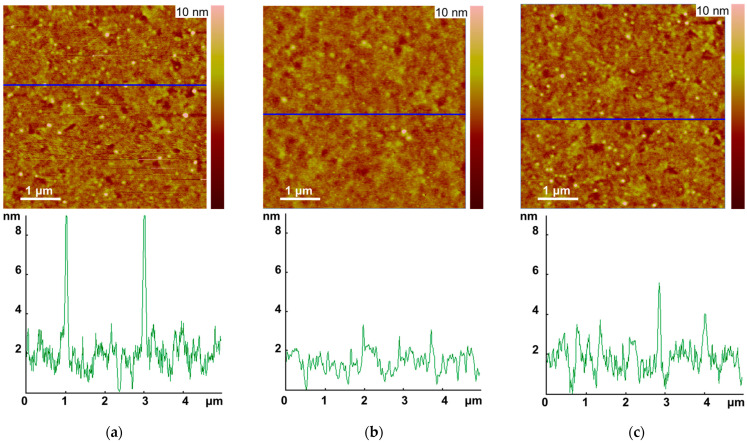
AFM images of the surface topography and sections corresponding to marked lines for (**a**) poly-TPD:PCBM, (**b**) poly-TPD:Cu-PP, and (**c**) poly-TPD:PCBM:Cu-PP composite films.

Surfaces of the 80 nm thick composite layers were characterized by AFM (Appendix B). The AFM provides reasonable resolution in z-direction for the surface topography of the studied samples (Figure 5).

The surface image of the ternary composite layer resembles a superposition of the poly-TPD:PCBM and poly-TPD:CuPP images. The surface topography of all layers is quite smooth with the root mean square roughness (RMS) not exceeding 2 nm: 0.8–1.1 nm for the polyTPD:PCBM, 0.8–1.0 nm for the polyTPD:PCBM:CuPP, and 0.6–0.9 nm for the polyTPD:CuPP sample (95% confidence intervals for six measurements). However, in the poly-TPD:PCBM topography, spherical inclusions (up to 10 nm in height) are clearly visible, which are actually invisible in the poly-TPD:CuPP image. The addition of PCBM to a polymer composite with a rough surface layer usually promotes a reduction in the RMS value. Yet, for a polymer composite with a smooth surface layer, the addition of PCBM can lead to the opposite result due to the formation of fullerene clusters in the composite layer [29].

### 3.3. Charge Carrier Mobility

In thin films of the studied composites, the mobility of charge carriers measured by the CELIV method is on the orders of 10^−5^ and 10^−4^ cm^2^V^−1^s^−1^ (Table 1). The poly-TPD:PCBM:Cu-PP ternary composite possesses the highest electron mobility and improved balance in electron and hole mobility compared to the other two composites.

Due to the small HOMO energy offsets between the donor poly-TPD and Cu-PP and the small LUMO energy offsets between the acceptor PCBM and Cu-PP (Figure 2b), Cu-PP molecules can provide the transport of both holes and electrons.

The hole mobility in the poly-TPD:Cu-PP composite is higher than in other composites and slightly lower than in neat poly-TPD. The Cu-PP molecule cannot act as a hole trap in the polymer matrix because its HOMO level is only slightly lower than that of poly-TPD (Figure 2b). However, Cu-PP molecules can influence the spatial arrangement of poly-TPD macromolecule fragments, making it difficult to transfer holes between them. Cu-PP molecules can also increase the width of the density of states (DOS) *σ* in poly-TPD:Cu-PP compared to that in neat poly-TPD. An increase in σ leads to a decrease in mobility according to expression (1) obtained in the Gaussian disorder model and the correlated disorder model to describe the charge transport properties of organic disordered semiconductors [2,30,31,32].

(1) $$\mu =\mu_{0}exp\left[-{\left(3\sigma /5kT\right)}^{2}+C_{0}\left({\left(\sigma /kT\right)}^{3/2}-\Gamma \right)\sqrt{qRF/\sigma }\right]$$

where *C*_0_ = 0.78, Γ = 2, *µ*_0_ is the pre-exponential factor, *R* is the distance between transport sites, *k* is the Boltzmann constant, *T* is absolute temperature, *q* is the elementary charge, *F* is the electric field strength.

The electron mobilities in the ternary composite and in the poly-TPD:PCBM composite are approximately an order of magnitude lower than in a neat PCBM. This is due to the high mobility of electrons in the latter. The electron mobility in the poly-TPD:PCBM composite is approximately two times higher than that in the poly-TPD:Cu-PP composite. A different effect of Cu-PP and PCBM on the electron mobility in poly-TPD composites may be associated with the different dispersion of these molecules in the poly-TPD matrix. It is well known that a mixture of a donor polymer (D) and an acceptor fullerene (A) forms a BHJ, the concept that was proposed for polymer solar cells about three decades ago [33,34]. In a three-dimensional BHJ, molecules D and A form a bicontinuous layer consisting of domains D and A. The interpenetrating network of the domains provides both a greater D–A interface for effective exciton dissociation and two channels for transporting electrons and holes to the corresponding electrodes.

In the AFM images (Figure 5), spherical objects representing fullerene clusters are clearly visible on the surface of the poly-TPD:PCBM and poly-TPD:PCBM:Cu-PP films, respectively. In contrast, on the poly-TPD:Cu-PP film, such objects are invisible. Thus, in contrast to PCBM molecules, Cu-PP molecules do not form a network of domains in the poly-TPD matrix; therefore, they are dispersed in the free volume of the polymer. For the homogeneous dispersion of the Cu-PP molecules in poly-TPD, the average intermolecular distance *R* estimated according to Equation (2) is equal to 1.48 nm, where *M* = 610 g/mole is the molecular weight of Cu-PP, *ρ* ≈ 1.3 g × cm^−3^ is the density of the solid film, *c* = 0.242 is the weight concentration of Cu-PP in poly-TPD and *N_A_* is the Avogadro number. This distance is quite sufficient to implement the transport of charge carriers through the mechanism of intermolecular hopping. Since PCBM molecules in domains are evidently located close to each other, the electron mobility in the poly-TPD:PCBM is higher than in poly-TPD:Cu-PP.

(2) $$R=\sqrt[{3}]{M/\rho cN_{A}}$$

In the ternary mixture, the Cu-PP molecules serve as electron transporting centers and thereby facilitate the transfer of electrons from separately located PCBM clusters to a continuous network of PCBM domains.

It is noteworthy that the electron mobility in PCBM, measured by us in the CELIV mode, is lower than that calculated using *J*–*V* characteristics of field-effect transistors (FETs): 4.5 × 10^−3^ cm^2^V^−1^s^−1^ [35] and 9 × 10^−3^ cm^2^V^−1^s^−1^ [36]. For hole mobility in neat poly-TPD, the same correlation is observed: the CELIV measurement shows a value of 2.8 × 10^−4^ (Table 1), which is an order of magnitude less than the FET mobility 2 × 10^−3^ [37]. Measurements with these two methods exhibit that there always seems to be about one order of magnitude difference in FET and CELIV mobilities for polymer and organic semiconductors [38,39].

In the studied poly-TPD composites, the mobility of electrons and holes, calculated from the *J*–*V* curves in the SCLC mode (Table 1), changes with changes in the additives to polymer in the same way as the CELIV mobility does (Table 2). However, to establish a generalized correlation between the absolute values of CELIV mobility and SCLC mobility, a wide array of experimental data is required.

### 3.4. Photoconductivity

**Figure 6:**
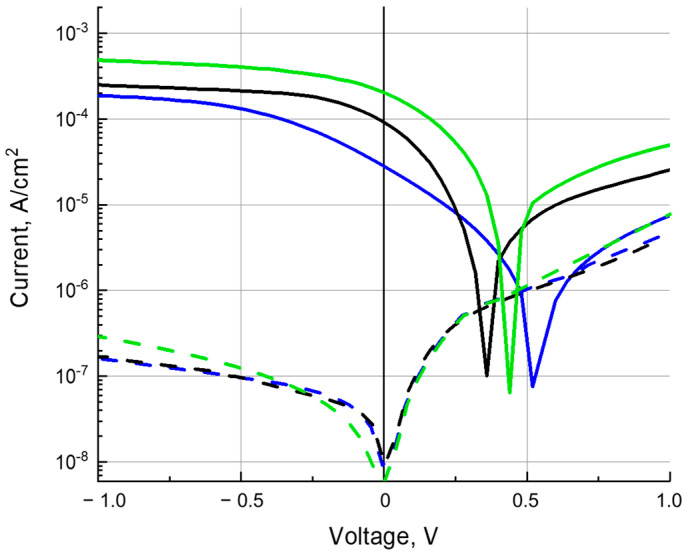
*J*–*V* curves of the dark current (dashed line) and photocurrent (solid line) under white light illumination for poly-TPD:Cu-PP- (blue), polyTPD:PCBM- (black), and poly-TPD:PCBM:Cu-PP- (green) based devices.

**Figure 7:**
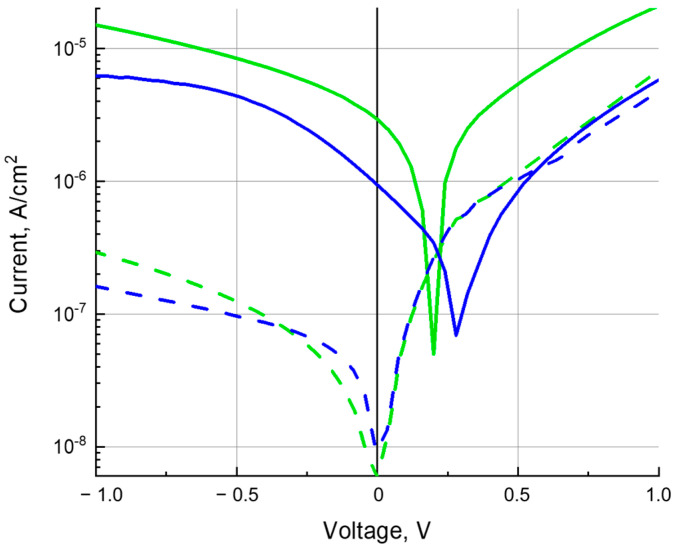
*J*–*V* curves of the dark current (dashed line) and photocurrent (solid line) under illumination at a wavelength of 650 nm (a power of 3 mW × cm^−2^) for poly-TPD:Cu-PP- (blue) and poly-TPD:PCBM:Cu-PP- (green) based devices.

The measurements of the *J*–*V* characteristics for ITO/PEDOT:PSS/composite/C_60_/BCP/Al diode devices (Figure 3) were carried out in the dark and under illumination with white light from a Xe lamp and at a wavelength of 650 nm at the Cu-PP absorption maximum. Presented in Figure 6 and Figure 7, *J*–*V* curves are typical characteristics of a photodiode. Under white light illumination (Figure 6) at a reverse bias of 1 V, the ratio of photo-to-dark current was about 1100, 1300, and 1500 for poly-TPD:Cu-PP (blue curves), poly-TPD:PCBM (black curves), and poly-TPD:PCBM:Cu-PP (green curves) based devices, respectively. Under illumination at a wavelength of 650 nm, (Figure 7), the ratio of photo-to-dark current was between 30 and 50 for Cu-PP-containing devices whereas the photocurrent was negligible in Cu-PP-free devices.

Photoconductivity σ_ph_ is expressed by the equation σ_ph_ = *q*(*µ*_e_ × *e + µ*_h_ × *p*), where *q* is the electron charge, *µ*_e_ and *µ*_h_ are the mobility of electrons and holes, respectively, *e* and *p* are the concentration of photogenerated electrons and holes, respectively. Additional absorption bands of Cu-PP in the red spectral band 590–690 nm and the charge transfer complex between Cu-PP and poly-TPD in the 450–500 nm (Figure 4c) band provide an increase in light harvesting and, as a consequence, an increase in *e* and *p*-values.

Due to the small HOMO energy offsets between the donor poly-TPD and Cu-PP and the small LUMO energy offsets between the acceptor PCBM and Cu-PP as seen in the energy diagram (Figure 2b), it is favorable for electrons and holes photogenerated on Cu-PP molecules to transfer to PCBM and poly-TPD, respectively. In turn, as shown in Section 3.3, the mobilities *µ*_e_ and *µ*_h_ are more balanced in the ternary composite than in the binary ones (Table 1 and Table 2). The strong mobility imbalance in the poly-TPD:Cu-PP film is evident in that the *J*–*V* curve increases slowly with increasing reverse bias compared to the *J*–*V* curves of the TPD:Cu-PP and TPD composites, which have a more balanced mobility (Figure 6 and Figure 7). If the mobilities of electrons and holes are approximately equal, then the probability of bimolecular recombination of charge carriers decreases, and, consequently, the loss of charge carrier concentration decreases.

The *J*–*V* curves show that the studied composites exhibit the photovoltaic effect. The open circuit voltage *V*_oc_ decreases in the sequence poly-TPD:Cu-PP-, poly-TPD:PCBM:Cu-PP-, and poly-TPD: PCBM-based devices. The result agrees with the empirical relation [40]:

(3) $$V_{OC}=\left(1/q\right)\left(\left|E_{HOMO}^{D}\right|-\left|E_{LUMO}^{A}\right|\right)-0.3V$$

here $E_{HOMO}^{D}$ is the HOMO level energy of the donor and $E_{LUMO}^{A}$ is the LUMO level energy of the acceptor. In (3), the *V_OC_* loss of 0.3 eV is empirical, and the loss could be greater or lesser. The reasons for voltage losses are still discussed [41,42].

The photoconductivity of the ternary composite demonstrates the promise of using the material in photodetector devices, in particular, those with sensitivity in the red spectral band. At the absorption maximum of Cu-PP, the external quantum efficiency (EQE) is equal to 0.5% for an incident flux of 1.87 × 10^16^ photons × s^−1^ × cm^−2^ at a wavelength of 650 nm and a photocurrent of 1.5 × 10^−5^ A × cm^−2^. In turn, the calculation of the internal quantum efficiency (IQE) gives a value of 5.7%, taking into account the decadic absorption coefficient (0.003 nm^−1^) of Cu-PP.

## 4. Conclusions

Based on the polymer poly-TPD having excellent thin film formation and hole transport abilities, we have developed a ternary composite with fullerene derivative PCBM and Cu-PP, a copper(II) complex with pyropheophorbide-a. We used the Cu-PP complex obtained from natural raw materials. The Cu-PP component blended with poly-TPD can (1) expand the photoconductivity of the composite in the blue–cyan and red bands of the spectrum, (2) serve as an electron acceptor with respect to the polymer in the same way as fullerene, and (3) provide the transport of both electrons and holes due to the small offset between HOMO levels of Cu-PP and poly-TPD and between LUMO levels of Cu-PP and PCBM. We focused on studying the charge transport. For the first time, the mobility of charge carriers in a poly-TPD composite with Cu-PP and in a ternary composite based on poly-TPD, Cu-PP, and PCBM was investigated, with a reasonably balanced mobility found in the ternary composite compared to the binary composites.

Based on charge mobility data and AFM topography images, we concluded that Cu-PP molecules are dispersed homogenously in the free volume of poly-TPD, while PCBM molecules form a network of domains in the polymer matrix (bulk heterojunction) serving as electron transporting pathways. Indeed, the electron mobility in poly-TPD:PCBM:Cu-PP is higher than in poly-TPD:PCBM, so the Cu-PP molecules, providing the hopping transport of electrons, can facilitate the transfer of electrons from separately dispersed PCBM clusters to the continuous network of the PCBM domains.

By blending poly-TPD with Cu-PP, the polymer composites are sensitized in the red spectral range and absorb more photons in the blue range. The results obtained on the photoconductivity of the composites demonstrate that the ternary composite is promising for use in photodetectors, in particular, those sensitive in the red range of the spectrum.

## Figures and Tables

**Table 1:** Charge carrier mobility in poly-TPD composites and individual components measured by the CELIV technique ^1^.

Composite	Mobility, cm^2^V^−1^s^−1^	
	Electrons	Holes
poly-TPD:PCBM	(8.0 ± 0.8) × 10^−5^	(1.4 ± 0.1) × 10^−4^
poly-TPD:Cu-PP	(3.5 ± 0.5) × 10^−5^	(2.2 ± 0.1) × 10^−4^
poly-TPD:PCBM:Cu-PP	(1.0 ± 0.1) × 10^−4^	(1.5 ± 0.1) × 10^−4^
poly-TPD	-	(2.8 ± 0.2) × 10^−4^
PCBM	(8.0 ± 0.8) × 10^−4^	-
Cu-PP	(6.0 ± 0.6) × 10^−5^	(4.5 ± 0.5) × 10^−5^

^1^ Calculated from 10 replicates, the confidence level is 90%.

**Table 2:** Charge carrier mobility in poly-TPD composite layers measured in the SCLC mode ^1^.

Composite	Mobility, cm^2^V^−1^s^−1^	
	Electrons	Holes
polyTPD:PCBM	(4.2 ± 0.5) × 10^−6^	(6.0 ± 0.6) × 10^−6^
polyTPD:Cu-PP	(1.9 ± 0.2) × 10^−6^	(7.0 ± 0.6) × 10^−6^
polyTPD:PCBM:Cu-PP	(8.3 ± 0.8) × 10^−6^	(9.8 ± 0.8) × 10^−6^

^1^ Calculated from 10 replicates, the confidence level is 90%.

## Data Availability

Data are contained within the article.

## Appendix B

AFM observations were carried out in the tapping mode (repulsive mode) in the air. EnviroScope (NanoScope5 controller) was used. Silicon cantilevers with spring constants of 5–40 N × m^−1^ and resonant frequencies of 150–350 kHz were used. During the measurement process, we made sure that the static charge did not blur the image. The measurements were carried out directly on working samples between the electrodes.
